# Heart Failure With Preserved Ejection Fraction and Adipose Tissue: A Story of Two Tales

**DOI:** 10.3389/fcvm.2019.00110

**Published:** 2019-08-02

**Authors:** Albin Oh, Ross Okazaki, Flora Sam, Maria Valero-Muñoz

**Affiliations:** ^1^Evans Department of Medicine, Boston Medical Center, Boston, MA, United States; ^2^Boston University School of Medicine, Boston, MA, United States; ^3^Section of Cardiovascular Medicine, Boston Medical Center, Boston, MA, United States; ^4^Whitaker Cardiovascular Institute, Boston University School of Medicine, Boston, MA, United States

**Keywords:** HFpEF, adipose tissue, obesity, natriuretic peptides, cardiac remodeling

## Abstract

Heart failure with preserved ejection fraction (HFpEF) is characterized by signs and symptoms of heart failure in the presence of a normal left ventricular ejection fraction. Although it accounts for up to 50% of all clinical presentations of heart failure, there are no evidence-based therapies for HFpEF to reduce morbidity and mortality. Additionally there is a lack of mechanistic understanding about the pathogenesis of HFpEF. HFpEF is associated with many comorbidities (such as obesity, hypertension, type 2 diabetes, atrial fibrillation, etc.) and is coupled with both cardiac and extra-cardiac abnormalities. Large outcome trials and registries reveal that being obese is a major risk factor for HFpEF. There is increasing focus on investigating the link between obesity and HFpEF, and the role that the adipose tissue and the heart, and the circulating milieu play in development and pathogenesis of HFpEF. This review discusses features of the obese-HFpEF phenotype and highlights proposed mechanisms implicated in the inter-tissue communication between adipose tissue and the heart in obesity-associated HFpEF.

## Heart Failure With Preserved Ejection Fraction (HFpEF): a New Term for an Old Disease

Heart failure (HF) is a clinical syndrome caused by structural and functional abnormalities in the heart that impair the ability of the ventricles to fill or eject blood. The cardinal manifestations of HF are breathlessness, dyspnea and fatigue, which may lead to limited effort tolerance; and fluid retention, thus resulting in pulmonary congestion and/or peripheral edema (1, 2). HF is a leading cause of morbidity and mortality both in the United States and worldwide. As of 2012, 5.8 million Americans had HF with the number of individuals with HF projected to continue to increase in the next 20 years (3–5).

Segregating patients with HF by left ventricular (LV) ejection fraction (EF) is an important phenotypic marker as it indicates unique pathophysiological mechanisms and thus subsequent responses to therapy (6–8). Patients with clinical HF and normal or preserved EF represent a phenotype that is different from those with reduced EF (HFrEF). HFpEF is due to the inability of the heart to fill with blood because it may be thick or stiff. HFpEF patients are often touted as elderly, predominantly female, obese, have long-standing hypertension, may have diabetes, and some degree of LV hypertrophy (9, 10). HFpEF was initially labeled as “diastolic HF” because impaired filling of the LV was thought to be the underlying etiology to differentiate it from “systolic HF” (HFrEF) (11). However, LV diastolic dysfunction is not unique to HFpEF and is also observed in patients with HFrEF (9, 12). Similarly, “diastolic HF” patients may have some degree of impaired systolic function (13, 14). Thus, the term “diastolic” HF was abandoned and replaced by HFpEF. The definition of HFpEF moved away from a primary focus on echocardiographic evidence of diastolic dysfunction, and toward a definition inclusive of cardiac structural abnormalities resulting from high filling pressures, diastolic abnormalities, elevated biomarkers, and increased left heart filling pressures by invasive hemodynamic measurements in the setting of an EF ≥50% (15–17).

In contrast to HFrEF, there are no evidence-based therapies, to date, which have shown improved outcomes in HFpEF (2), likely because of the marked heterogeneity of the HFpEF syndrome (16, 18). It has been suggested that phenotyping patients into pathophysiologically homogeneous groups in clinical trials may result in better outcomes (19–21). Increased adiposity in obesity has been suggested to be a therapeutic target in HFpEF (22). This review, therefore, summarizes the current understanding of HFpEF in context of obesity, and how “crosstalk” exists between the heart and the adipose tissue in these two conditions.

### The Obese-HFpEF Phenotype

Obesity has reached epidemic proportions worldwide and is a major comorbidity in HFpEF patients (23–25). The prevalence of being overweight and obese is as high as 84% in clinical trials, epidemiological studies and HF registries (26–28) and presently there are >1.8 million persons in the U.S. with an overweight or obesity-associated HFpEF phenotype (22). Earlier studies suggested that symptoms in obese HFpEF patients were simply related to excess body mass and not to cardiac abnormalities (29). However, recent disease paradigms have incorporated obesity into the pathophysiology of HFpEF (24). Obesity and related cardio-metabolic traits are also more strongly associated with the risk of future HFpEF rather than HFrEF (30), suggesting that obesity-associated HFpEF represents a distinct clinical phenotype within the broad spectrum of HFpEF (24, 31). Studies from murine models have highlighted the relationship between obesity, diastolic dysfunction and HFpEF. Increased adiposity and metabolic alterations in obesity were associated with cardiac structural remodeling and diastolic dysfunction in mice and rats (32, 33), and have recently been described to induce HFpEF (34–38). These models are useful tools to investigate mechanisms linking obesity and HFpEF and to explore the use of potential therapies in this specific phenotype (39). However, there is no animal model that can completely mimic the human disease, partly because human HFpEF is heterogeneous and encompasses a broad range of signs, symptoms, and disease presentation (39). Thus, the paucity of highly characterized HFpEF animal models that reflect cardiopulmonary and metabolic changes seen in obesity associated-HFpEF in humans contributes to the lack of understanding of the mechanisms underlying HFpEF and the development of treatments.

## The Adipose Tissue and the Heart Cross-Talk in HFpEF

There is an increasing appreciation of the complex connection between the adipose tissue and the heart, which highlights the importance of the heart-adipose-axis in the pathogenesis of cardiovascular disease and specifically HF (40). However, the putative mechanisms that connect both tissues and link obesity and HF have not been fully elucidated (23, 41). It was long assumed that the burden of obesity in HF was a physical/mechanical one (42). Thus, hemodynamic alterations that result from excessive adipose accumulation in obese patients would have subsequent effects on cardiac morphology and ventricular function (43). Although volume overload plays a role in HF and specifically HFpEF, in recent years, the endocrine, metabolic and cellular signaling behind the obesity-related HFpEF phenotype has received much attention.

Current evidence supports the hypothesis that obesity-related HFpEF may result from adipokines imbalance, neprilysin over-activity and/or augmented mineralocorticoid signaling (44). Adipose tissue is a potent endocrine organ that synthesizes and secretes a number of adipose-specific cytokines, *aka* adipokines, such as leptin or adiponectin, which elicit a variety of local and systemic responses (45). Leptin originates mainly from subcutaneous adipose tissue (46) and circulating levels of leptin directly correlate with fat mass in both obese rodents and humans (40). Leptin plays an important role in the regulation of the sympathetic nervous system, affecting heart rate and blood pressure (47) and exert its effects by activating various mediators including the Janus kinases (JAK)/Signal Transducer and Activator of Transcription proteins (STAT), the phosphoinositide 3-kinase (PI3K)/ cGMP-dependent protein kinase B (PKB) and the p38 mitogen-activated protein kinase (p38-MAPK) pathways (48). Alterations in leptin signaling have deleterious effects in cardiac remodeling in pre-clinical models of obesity (33). Additionally, leptin is a major stimulus for the production of aldosterone in obesity (49, 50), and might be responsible for the exacerbated mineralocorticoid receptor signaling in obesity-related HF (51, 52). In addition to aldosterone-mediated changes in cardiac structure, such as exacerbated cardiac remodeling (53, 54), increased leptin results in impaired calcium handling and impaired relaxation in the heart (55, 56). However, although the contribution of leptin to the genesis and progression of the obese-HFpEF phenotype has been speculated (42), there are no mechanistic or clinical evidences to support leptin's role in the HFpEF phenotype. In contrast to leptin, adiponectin levels are highest in lean subjects but decline as body mass increases (57). Adiponectin have multiple beneficial effects in the heart and the vasculature (45) and, not surprisingly, depressed levels in obesity are associated with inflammation and greater cardiovascular risk (58–60). Experimental evidence showed that adiponectin has anti-inflammatory properties (61) and modulates oxidative stress-induced autophagy (62) and cardiac remodeling (63). These beneficial effects of adiponectin have been linked to direct effects of this adipokine on the cellular in the heart and blood vessels. It has been postulated that the ability of adiponectin to attenuate cardiac hypertrophy and fibrosis is likely due to its ability to stimulate AMP-activated protein kinase (AMPK)-dependent and extracellular-signal-regulated kinase (ERK) signaling within cardiac myocytes and endothelial cells (63–65). However, although adiponectin levels are not predictive of HF development in humans (66), human studies indicate that elevated circulating adiponectin is associated with increased mortality in chronic HFrEF patients (67–69). These findings have been partly explained by the fact that adiponectin upregulation seems to be liked to cachexia and adiponectin raised levels may just reflect the hyper-catabolic state in severe HF (70, 71). This is consistent with the fact that overweight and obese HFrEF patients had normal levels of adiponectin (72). In contrast, circulating levels of adiponectin are markedly reduced in obese HFpEF patients, particularly in women (73), and it has been suggested that adiponectin may prevent some of the pathophysiologic mechanisms underlying the obese-HFpEF such as myocardial hypertrophy, cardiac fibrosis, oxidative stress, and inflammation (44, 60). The relationship of adiponectin to aldosterone appears to be polar opposite in HFpEF, as adiponectin deficiency in a preclinical model of hypertension-associated HFpEF where aldosterone is elevated, exacerbated cardiac remodeling, diastolic dysfunction and pulmonary congestion (74); and adiponectin overexpression protected against the progression of HFpEF by regulating oxidative stress and modulating calcium-handling proteins, specifically cAMP-dependent protein kinase (PKA) phosphorylation of phospholamban (75).

Chronic, low-grade inflammation is also a hallmark of obese adipose tissue (76) and systemic metabolic inflammation, accompanied by an increased activity of the inducible nitric oxide synthase (iNOS) and augmented nitrosative stress, may play an important role in the pathophysiology of obesity-associated HFpEF (77). This is supported by the hypothesis that imbalance in the nitrate-nitrite-nitric oxide pathway plays a role both in the peripheral abnormalities that contribute to HFpEF, such as increased arterial stiffness and abnormalities in skeletal muscle fiber type and capillary density (78). Increased oxidative stress in the coronary microvascular endothelium due to decreased nitric oxide bioavailability and reduced cGMP dependent protein kinase (PKG) activity in cardiac myocytes, results in increased cardiac stiffening and hypertrophy (5) thus contributing to the cardiac abnormalities. Additionally, the clinical relevance of proinflammatory cytokines in obesity-associated HFpEF is being actively investigated, with promising targets including inflammasome, toll-like receptors, cytokines and macrophages (79, 80). Notably, interleukin 1 (IL-1) has been strongly associated with adverse cardiac remodeling and heart failure and strategies targeting the IL-1 pathway are currently undergoing clinical evaluation (81, 82).

### Obesity and Exercise Tolerance in HFpEF

Decreased exercise tolerance is an early symptom of HFpEF and is a major determinant of prognosis and associates with a reduction in quality of life (83). Exercise capacity is defined as the rate of O_2_ consumption (VO_2_) at peak exercise, and any factor that limits peak VO_2_, by impeding O_2_ delivery and/or utilization, can cause exercise intolerance (84). Although exercise intolerance in HFpEF was classically attributed to changes in cardiac output, new findings suggest that peripheral, non-cardiac factors play an important role in the limitations in exercise capacity in patients with HFpEF (85). Of these, obesity has been also proposed to be a major driver of exercise intolerance, independent of the effects of cardiac function (86). Interestingly, the pattern of regional adipose deposition, with increased intra-abdominal and inter-muscular fat appear to associate with decreased peak VO_2_, and may thus be related to adverse consequences in exercise tolerance in HFpEF beyond total body adiposity (87).

It has been suggested that higher levels of exercise training may attenuate the increased risk of HF associated with obesity (88). Exercise, in addition to caloric restriction-induced weight loss, are the only interventions shown to improve exercise capacity outcome in HFpEF (89–92). Furthermore, a recent study demonstrated that exercise training improved not only exercise capacity but also body composition, with a reduction in total fat mass and thigh muscle/inter-muscular fat ratio, and with reduced inflammation and LV mass (92). Similarly, preclinical studies in obese HFpEF rats showed that exercise training improved exercise capacity (36). Further studies are warranted in order to investigate specific mechanisms involved.

### The Obesity Paradox

Although obesity is linked to the development of HF (23) and associates with abnormal hemodynamics and adverse cardiac remodeling in HFpEF (93), in epidemiological studies mild to moderate overweight or obesity status (body mass index, BMI, of 30-34.9) was reported to have a protective effect in patients with HF (94, 95). This phenomenon was termed “the obesity paradox” and initially observed in small population studies (96, 97) and confirmed in large observational studies in both HFrEF and HFpEF patients (26, 98–101). However, other studies have not shown that the obesity paradox exists in HFpEF (102–104), and thus, the causal link between this scientific observation and its clinical implications are limited and remain hotly debated. Several hypotheses are proposed to explain the presence or absence of the obesity paradox (105, 106), and have been extensively reviewed (107–109).

### Cardiac Natriuretic Peptides and Obesity in HFpEF

Cardiac natriuretic peptides are mainly released from the heart in response to myocardial stress and have a key role in cardiovascular homeostasis (110). There are three types of natriuretic peptides in humans, atrial natriuretic peptide (ANP), brain natriuretic peptide (BNP) and C-type natriuretic peptide (CNP). ANP and BNP are released from the atria and ventricles of the heart respectively and are the most physiologically active natriuretic peptides. In contrast, CNP is thought to act locally, as a paracrine/autocrine regulator, since it is cleared rapidly from the circulation and present at very low concentrations in plasma (111) with effects primarily on bone growth (112). ANP and BNP bind to two homodimeric receptors, natriuretic peptide active and clearance receptors (NPRA and NPRC respectively), which are expressed in many tissues, including white and brown adipose tissue (113). This broad distribution is indicative of the wide biological effects of the natriuretic peptides. Although ANP and BNP were initially characterized by their actions promoting diuresis and natriuresis, contributing to maintenance of extracellular fluid volume and vascular tone (114), they mediate actions beyond simply control of blood pressure and volume homeostasis. These include but are not limited to obesity and metabolic regulation, atherosclerotic and thrombotic control, and cardiac remodeling (115).

ANP and BNP are synthesized as precursor pro-hormones (proANP and proBNP) which are then processed to their biologically active forms ANP and BNP, and biologically inactive N-terminal proANP (NT-proANP) and NT-proBNP forms (116). Of these, BNP and NT-proBNP have demonstrated diagnostic and prognostic value in patients with HF (117). Increased BNP is independently associated with the increased risk of developing HF even within an asymptomatic general population (118) and once HF manifests, higher BNP levels are associated with increased risk of adverse events (119). Whereas, BNP and NT-ProBNP are elevated in clinical HF regardless of the LV EF, these levels are usually higher in HFrEF than in HFpEF (120, 121). Circulating BNP levels are also typically lower in patients with obesity compared to normal weight counterparts given a similar degree of clinical HF. This is evident in HFpEF, where obese patients with HFpEF usually have lower circulating BNP and NT-ProBNP levels than non-obese patients (24, 122). However, despite the reduced levels of natriuretic peptides in obese patients, they still serve as an important tool in HF both for screening and prognostic purposes, albeit at a lower threshold (93, 123, 124). Obesity in mice and rats is associated with a reduction in natriuretic peptides levels (125, 126), even in the setting of impaired cardiac function (127).

### The Natriuretic Handicap

The inverse relationship between circulating cardiac BNP and obesity (defined by BMI) is termed the “natriuretic handicap” and has been described in both healthy subjects and patients with HF (31, 128). It has been hypothesized that BNP levels are reduced in obesity due to the differential expression of their clearance receptor (NPRC) resulting in enhanced degradation in adipose tissue (129). Additionally, others showed that obese patients have decreased natriuretic peptides production (130, 131); consistent with pre-clinical studies in murine obesity models showing reduced levels of natriuretic peptides cardiac mRNA expression (126, 132). Other mechanisms linking natriuretic peptides reduction and insulin resistance have also been proposed to explain this inverse relationship (133, 134). ANP and BNP can be also degraded by extracellular proteases such as neprilysin (116, 135). Neprilysin is secreted by adipocytes and promotes adipogenesis, creating a positive feedback loop. People with obesity have increased levels of neprilysin in proportion with their body mass (136) and neprilysin levels are particularly elevated in obese patients with HFpEF (137). NT-proBNP is mainly cleared by renal excretion and is not a substrate for neprilysin degradation (138). A recent phase II clinical trial investigated the effect of an angiotensin receptor neprilysin inhibitor (LCZ696) in overweight/obese HFpEF patients for 36 weeks and found left atrial reverse remodeling and improvement in NYHA class. These results were accompanied with a reduction in NT-proBNP suggesting that LCZ696 reduced left ventricular pressures and wall stress (139), and provided the rationale for an outcomes trial in HFpEF, which is presently underway (140).

### Cardiac Natriuretic Peptides Signaling in the Adipose Tissue

**Figure 1:**
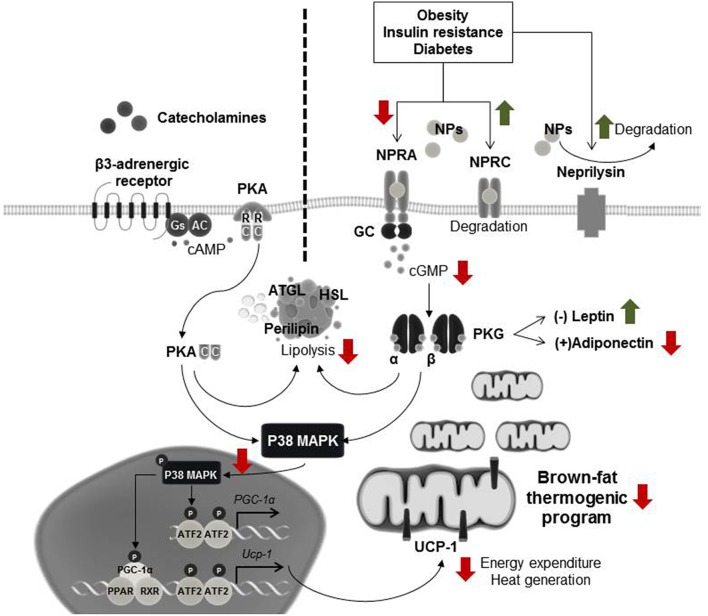
Natriuretic peptide signaling in adipose tissue. Cardiac stress, such as HFpEF, induces increased natriuretic peptides levels. These natriuretic peptides bind to their receptor, natriuretic peptide active receptor (NPRA), in the adipocyte, and activate guanylyl cyclase (GC), increasing cGMP levels. Adipocytes also express natriuretic peptide clearance receptor (NPRC) that functions to remove natriuretic peptides from the circulation. The cGMP produced by NPRA-GC activates cGMP dependent protein kinase (PKG), which triggers a signaling cascade that results in enhanced lipolysis and activation of p38 mitogen-activated protein kinase (p38-MAPK), culminating in the transcription of uncoupling protein 1 (UCP-1) and inducing the brown fat thermogenic program. In parallel, other stimuli, such as cold exposure, can also induce this program via the β-adrenergic signaling pathway. Here catecholamines bind to the β-adrenergic receptor which activates adenylate cyclase (AC), producing cAMP. Binding of cAMP to the regulatory subunits (R) of cAMP-dependent protein kinase (PKA) releases its catalytic subunits (C), which also activate lipolysis and induce p38-MAPK phosphorylation. During obesity, insulin resistance and diabetes, the natriuretic peptide signaling is diminished leading to a decrease in the browning thermogenic program. Red and green arrows represent the down-regulatory or up-regulatory effects that metabolic disorders have in this signaling pathway. To date, the combined effect that obesity and HFpEF would have in adipose tissue is unknown and needs further investigation.

White adipose tissue was previously thought to only function as an energy storage unit with limited metabolic activity, and human brown adipose tissue to be active only in infants before disappearing in childhood. It is now known that both, white and brown adipose tissues have in highly active roles in metabolic regulation (141–143). We and others recently showed that cardiac natriuretic peptide signaling causes alterations in energy expenditure and metabolism, and promotes brown adipose-like features in white adipose tissue depots (144–147) and that this is evident in HFpEF (146). Natriuretic peptide signaling is mediated predominantly through the binding of NPRA, which possesses intrinsic guanylyl cyclase activity. Conversely, NPRC serves primarily as the clearance receptor, sequestering natriuretic peptides from the circulation for internalization and subsequent degradation (112). Thus, the ratio of NPRA to NPRC is an important regulator of overall natriuretic peptide activity (148). Upon binding of natriuretic peptides to NPRA in the adipocyte, the receptor's guanylyl cyclase is activated, producing cGMP, which then activates intracellular PKG (112, 149). PKG phosphorylates several lipolytic proteins, including hormone-sensitive lipase (HSL), perilipin, and adipose triglyceride lipase (ATGL), resulting in the breakdown of stored lipids into free fatty acids. In parallel, PKG phosphorylates p38-MAPK, which modulates the brown-fat thermogenic program by increasing transcription of proteins such as uncoupling protein-1 (UCP-1) and peroxisome proliferator activated receptor gamma coactivator 1 alpha (PGC-1α) (146, 149). UCP-1 is responsible for the uncoupling of oxidative phosphorylation and PGC-1α is the key regulator of oxidative metabolism (141, 150). UCP1 and PGC-1α promote mitochondrial biogenesis and coupled and uncoupled respiration resulting in enhanced energy expenditure and thereby limiting adipose tissue expansion (110). Natriuretic peptide signaling in adipose tissue shares activity homology and similar potency with sympathetic activation via β-adrenergic receptors (145). Sympathetic stimulators, such as cold temperature, increase circulating catecholamines that bind to β-adrenergic receptors on adipose tissue (151–153). This increases PKA via a cAMP-dependent mechanism. PKA shares homology with PKG thus both sympathetic nervous-system and natriuretic peptide signaling increase metabolic activity in adipose tissue by activating lipolysis, and modulating the brown-fat thermogenic program through p38-MAPK (113, 147, 149) (Figure 1).

Metabolic disorders such as obesity and type 2 diabetes are associated with dysregulation of the natriuretic peptide system (154, 155). The natriuretic peptide receptor ratio in adipose tissue was inversely associated with obesity, glucose intolerance and insulin resistance in a cross-sectional analysis of subjects with a wide range of BMI and glucose tolerance (156). Insulin, which modulates blood glucose levels, exerts potent lipogenic effects, and is also an important regulator of natriuretic peptide activity. A low insulin fasting-state leads to an increase in NPRA mRNA and a decrease in NPRC mRNA whereas conversely, in hyperinsulinemic ob/ob mice, levels of NPRC mRNA are increased and levels of NPRA mRNA are decreased (157, 158). Similarly NPRA mRNA levels are lower in human adipocytes obtained from individuals with pre-diabetes and type 2 diabetes. Treatment with BNP also increases glucose uptake in adipose tissue independent of insulin levels. This is mediated via PKB phosphorylation and the mechanistic target of rapamycin complex (mTORC)1/2 activation, leading to translocation of glucose transporter 4 (GLUT4) to the cell membrane (159). Thus, insulin inhibits natriuretic peptides, while natriuretic peptides increase insulin sensitivity and help to control blood glucose levels.

There is also interplay between natriuretic petides released from the heart and adipokines released by adipose tissue. ANP decreases the secretion of leptin in cultured human subcutaneous adipose tissue (160) and isolated human adipocytes from obese individuals (161). An inverse relationship between circulating BNP and plasma levels of leptin also exists in HFrEF patients (162). Yet, adiponectin synthesis and secretion has been positively associated with natriuretic peptides. ANP acutely increased systemic levels of adiponectin in healthy subjects (163) and both, ANP and BNP, promoted the expression and secretion of adiponectin in human adipocytes in culture and in chronic HFrEF patients (164). These findings are also consistent with observational studies showing positive associations between circulating levels of adiponectin and BNP in healthy subjects without HF (165) and HFrEF patients (67). Thus, higher adiponectin levels tend to be associated with reduced LV systolic function in humans (166).

## Concluding Remarks

HFpEF is a major public problem that is increasing in prevalence yet lacking in evidence-based therapies. A more tailored approach in HFpEF is needed to investigate the pathophysiological mechanisms that underlie this syndrome. Obesity-associated HFpEF is an important sub-phenotype of HFpEF, with evidence supporting crosstalk between the heart and the adipose tissue. Thus, the ability to modulate the signaling pathways that regulate adipose tissue and the heart in HFpEF might have clinical implications and be translated into effective therapies for HFpEF, particularly obesity-associated HFpEF.

## Author Contributions

FS and MV-M discussed and conceived the outline of the manuscript. AO, RO, and MV-M drafted the initial version of the manuscript. FS and MV-M reviewed the manuscript. All authors approved the final version of the manuscript.

### Conflict of Interest Statement

The authors declare that the research was conducted in the absence of any commercial or financial relationships that could be construed as a potential conflict of interest.

## References

[1] Metra M Teerlink JR. Heart failure. Lancet. (2017) 390:1981–95. 10.1016/S0140-6736(17)31071-1 28460827

[2] Ponikowski P Voors AA Anker SD Bueno H Cleland JG Coats AJ. 2016 ESC Guidelines for the diagnosis and treatment of acute and chronic heart failure: the Task Force for the diagnosis and treatment of acute and chronic heart failure of the European Society of Cardiology (ESC). Developed with the special contribution of the Heart Failure Association (HFA) of the ESC. Eur J Heart Fail. (2016) 37:2129–200. 10.1093/eurheartj/ehw128 27206819

[3] Benjamin EJ Muntner P Alonso A Bittencourt MS Callaway CW Carson AP. Heart disease and stroke statistics-2019 update: a report from the American Heart Association. Circulation. (2019) 139:e56–28. 10.1161/CIR.0000000000000659 30700139

[4] Heidenreich PA Albert NM Allen LA Bluemke DA Butler J Fonarow GC. Forecasting the impact of heart failure in the United States: a policy statement from the American Heart Association. Circ Heart Fail. (2013) 6:606–19. 10.1161/HHF.0b013e318291329a 23616602 PMC3908895

[5] Paulus WJ Tschope C. A novel paradigm for heart failure with preserved ejection fraction: comorbidities drive myocardial dysfunction and remodeling through coronary microvascular endothelial inflammation. J Am Coll Cardiol. (2013) 62:263–71. 10.1016/j.jacc.2013.02.092 23684677

[6] Sanderson JE. HFNEF, HFpEF, HF-PEF, or DHF: what is in an acronym? JACC Heart Fail. (2014) 2:93–4. 10.1016/j.jchf.2013.09.006 24622122

[7] McMurray JJ. Clinical practice. Systolic heart failure. N Engl J Med. (2010) 362:228–38. 10.1056/NEJMcp0909392 20089973

[8] Redfield MM. Heart failure with preserved ejection fraction. N Engl J Med. (2016) 375:1868–77. 10.1056/NEJMcp1511175 27959663

[9] Sanderson JE. Heart failure with a normal ejection fraction. Heart. (2007) 93:155–8. 10.1136/hrt.2005.074187 16387829 PMC1861394

[10] Beale AL Meyer P Marwick TH Lam CSP Kaye DM. Sex Differences in cardiovascular pathophysiology: why women are overrepresented in heart failure with preserved ejection fraction. Circulation. (2018) 138:198–205. 10.1161/CIRCULATIONAHA.118.034271 29986961

[11] Hunt SA Baker DW Chin MH Cinquegrani MP Feldman AM Francis GS. ACC/AHA guidelines for the evaluation and management of chronic heart failure in the adult: executive summary. A report of the American College of Cardiology/American Heart Association Task Force on Practice Guidelines (Committee to revise the 1995 Guidelines for the Evaluation and Management of Heart Failure). J Am Coll Cardiol. (2001) 104:2996–3007. 10.1161/hc4901.102568 11738322

[12] McMurray J Pfeffer MA. New therapeutic options in congestive heart failure: Part II. Circulation. (2002) 105:2223–8. 10.1161/01.CIR.0000014771.38666.22 11994259

[13] Tan YT Wenzelburger F Lee E Heatlie G Leyva F Patel K. The pathophysiology of heart failure with normal ejection fraction: exercise echocardiography reveals complex abnormalities of both systolic and diastolic ventricular function involving torsion, untwist, and longitudinal motion. J Am Coll Cardiol. (2009) 54:36–46. 10.1016/j.jacc.2009.03.037 19555838

[14] Borlaug BA Olson TP Lam CS Flood KS Lerman A Johnson BD. Global cardiovascular reserve dysfunction in heart failure with preserved ejection fraction. J Am Coll Cardiol. (2010) 56:845–54. 10.1016/j.jacc.2010.03.077 20813282 PMC2950645

[15] Prasad A Hastings JL Shibata S Popovic ZB Arbab-Zadeh A Bhella PS. Characterization of static and dynamic left ventricular diastolic function in patients with heart failure with a preserved ejection fraction. Circ Heart Fail. (2010) 3:617–26. 10.1161/CIRCHEARTFAILURE.109.867044 20682947 PMC3716372

[16] Sharma K Kass DA. Heart failure with preserved ejection fraction: mechanisms, clinical features, and therapies. Circ Res. (2014) 115:79–96. 10.1161/CIRCRESAHA.115.302922 24951759 PMC4146618

[17] Yancy CW Jessup M Bozkurt B Butler J Casey DE Jr Colvin MM. 2017 ACC/AHA/HFSA Focused Update of the 2013 ACCF/AHA guideline for the management of heart failure: a report of the American College of Cardiology/American Heart Association Task Force on clinical practice guidelines and the heart failure society of America. J Am Coll Cardiol. (2017) 136:e137-61. 10.1161/CIR.0000000000000509 28461007

[18] Roh J Houstis N Rosenzweig A. Why don't we have proven treatments for HFpEF? Circ Res. (2017) 120:1243–5. 10.1161/CIRCRESAHA.116.310119 28408453 PMC5407384

[19] Shah SJ Katz DH Selvaraj S Burke MA Yancy CW Gheorghiade M. Phenomapping for novel classification of heart failure with preserved ejection fraction. Circulation. (2015) 131:269–79. 10.1161/CIRCULATIONAHA.114.010637 25398313 PMC4302027

[20] Shah SJ Kitzman DW Borlaug BA van HL Zile MR Kass DA. Phenotype-specific treatment of heart failure with preserved ejection fraction: a multiorgan roadmap. Circulation. (2016) 134:73–90. 10.1161/CIRCULATIONAHA.116.021884 27358439 PMC4930115

[21] Borlaug BA. The pathophysiology of heart failure with preserved ejection fraction. Nat Rev Cardiol. (2014) 11:507–15. 10.1038/nrcardio.2014.83 24958077

[22] Kitzman DW Shah SJ. The HFpEF obesity phenotype: the elephant in the room. J Am Coll Cardiol. (2016) 68:200–3. 10.1016/j.jacc.2016.05.019 27386774

[23] Kenchaiah S Evans JC Levy D Wilson PW Benjamin EJ Larson MG. Obesity and the risk of heart failure. N Engl J Med. (2002) 347:305–13. 10.1056/NEJMoa020245 12151467

[24] Obokata M Reddy YNV Pislaru SV Melenovsky V Borlaug BA. Evidence Supporting the existence of a distinct obese phenotype of heart failure with preserved ejection fraction. Circulation. (2017) 136:6–19. 10.1161/CIRCULATIONAHA.116.026807 28381470 PMC5501170

[25] Tsujimoto T Kajio H. Abdominal Obesity is associated with an increased risk of all-cause mortality in patients with HFpEF. J Am Coll Cardiol. (2017) 70:2739–49. 10.1016/j.jacc.2017.09.1111 29191321

[26] Haass M Kitzman DW Anand IS Miller A Zile MR Massie BM. Body mass index and adverse cardiovascular outcomes in heart failure patients with preserved ejection fraction: results from the Irbesartan in Heart Failure with Preserved Ejection Fraction (I-PRESERVE) trial. Circ Heart Fail. (2011) 4:324–31. 10.1161/CIRCHEARTFAILURE.110.959890 21350053 PMC3100162

[27] Redfield MM Chen HH Borlaug BA Semigran MJ Lee KL Lewis G. Effect of phosphodiesterase-5 inhibition on exercise capacity and clinical status in heart failure with preserved ejection fraction: a randomized clinical trial. JAMA. (2013) 309:1268–77. 23478662 10.1001/jama.2013.2024 PMC3835156

[28] Anjan VY Loftus TM Burke MA Akhter N Fonarow GC Gheorghiade M. Prevalence, clinical phenotype, and outcomes associated with normal B-type natriuretic peptide levels in heart failure with preserved ejection fraction. Am J Cardiol. (2012) 110:870–6. 10.1016/j.amjcard.2012.05.014 22681864 PMC3432159

[29] Caruana L Petrie MC Davie AP McMurray JJ. Do patients with suspected heart failure and preserved left ventricular systolic function suffer from “diastolic heart failure” or from misdiagnosis? A prospective descriptive study. BMJ. (2000) 321:215–8. 10.1136/bmj.321.7255.215 10903655 PMC27439

[30] Savji N Meijers WC Bartz TM Bhambhani V Cushman M Nayor M. The Association of Obesity and Cardiometabolic Traits With Incident HFpEF and HFrEF. JACC Heart Fail. (2018) 6:701–9. 10.1016/j.jchf.2018.05.018 30007554 PMC6076337

[31] Clerico A Zaninotto M Passino C Plebani M. Obese phenotype and natriuretic peptides in patients with heart failure with preserved ejection fraction. Clin Chem Lab Med. (2018) 56:1015–25. 10.1515/cclm-2017-0840 29381470

[32] Bostick B Habibi J DeMarco VG Jia G Domeier TL Lambert MD. Mineralocorticoid receptor blockade prevents Western diet-induced diastolic dysfunction in female mice. Am J Physiol Heart Circ Physiol. (2015) 308:H1126-H1135. 10.1152/ajpheart.00898.2014 25747754 PMC4551127

[33] Martinez-Martinez E Jurado-Lopez R Valero-Munoz M Bartolome MV Ballesteros S Luaces M. Leptin induces cardiac fibrosis through galectin-3, mTOR and oxidative stress: potential role in obesity. J Hypertens. (2014) 32:1104–14. 10.1097/HJH.0000000000000149 24695395

[34] Alex L Russo I Holoborodko V Frangogiannis NG. Characterization of a mouse model of obesity-related fibrotic cardiomyopathy that recapitulates features of human heart failure with preserved ejection fraction. Am J Physiol Heart Circ Physiol. (2018) 315:H934–49. 10.1152/ajpheart.00238.2018 30004258 PMC6230908

[35] Liu Y Li LN Guo S Zhao XY Liu YZ Liang C. Melatonin improves cardiac function in a mouse model of heart failure with preserved ejection fraction. Redox Biol. (2018) 18:211–21. 10.1016/j.redox.2018.07.007 30031269 PMC6076208

[36] Bowen TS Brauer D Rolim NPL Baekkerud FH Kricke A Ormbostad Berre AM. Exercise training reveals inflexibility of the diaphragm in an animal model of patients with obesity-driven heart failure with a preserved ejection fraction. J Am Heart Assoc. (2017) 6:e006416. 10.1161/JAHA.117.006416 29066440 PMC5721851

[37] Schmederer Z Rolim N Bowen TS Linke A Wisloff U Adams V. Endothelial function is disturbed in a hypertensive diabetic animal model of HFpEF: moderate continuous vs. high intensity interval training. Int J Cardiol. (2018) 273:147–54. 10.1016/j.ijcard.2018.08.087 30193792

[38] Meng Q Lai YC Kelly NJ Bueno M Baust JJ Bachman TN. Development of a mouse model of metabolic syndrome, pulmonary hypertension, and heart failure with preserved ejection fraction. Am J Respir Cell Mol Biol. (2017) 56:497–505. 10.1165/rcmb.2016-0177OC 28118022 PMC5449511

[39] Valero-Munoz M Backman W Sam F. Murine models of heart failure with preserved ejection fraction: a “Fishing Expedition”. JACC Basic Transl Sci. (2017) 2:770–89. 10.1016/j.jacbts.2017.07.013 29333506 PMC5764178

[40] Turer AT Hill JA Elmquist JK Scherer PE. Adipose tissue biology and cardiomyopathy: translational implications. Circ Res. (2012) 111:1565–77. 10.1161/CIRCRESAHA.111.262493 23223931 PMC3532954

[41] Massie BM Obesity and heart failure–risk factor or mechanism? N Engl J Med. (2002) 347:358–9. 10.1056/NEJMe020065 12151474

[42] Packer M. Leptin-aldosterone-neprilysin axis: identification of its distinctive role in the pathogenesis of the three phenotypes of heart failure in people with obesity. Circulation. (2018) 137:1614–31. 10.1161/CIRCULATIONAHA.117.032474 29632154

[43] Alpert MA Lavie CJ Agrawal H Aggarwal KB Kumar SA. Obesity and heart failure: epidemiology, pathophysiology, clinical manifestations, and management. Transl Res. (2014) 164:345–56. 10.1016/j.trsl.2014.04.010 24814682

[44] Packer M Kitzman DW. Obesity-Related Heart Failure With a Preserved Ejection Fraction: The mechanistic rationale for combining inhibitors of aldosterone, neprilysin, and sodium-glucose cotransporter-2. JACC Heart Fail. (2018) 6:633–9. 10.1016/j.jchf.2018.01.009 29525327

[45] Akoumianakis I Antoniades C. The interplay between adipose tissue and the cardiovascular system: is fat always bad? Cardiovasc Res. (2017) 113:999–1008. 10.1093/cvr/cvx111 28582523

[46] Kershaw EE Flier JS. Adipose tissue as an endocrine organ. J Clin Endocrinol Metab. (2004) 89:2548–56. 10.1210/jc.2004-0395 15181022

[47] Correia ML Morgan DA Sivitz WI Mark AL Haynes WG. Leptin acts in the central nervous system to produce dose-dependent changes in arterial pressure. Hypertension. (2001) 37:936–42. 10.1161/01.HYP.37.3.936 11244021

[48] Fruhbeck G. Intracellular signalling pathways activated by leptin. Biochem J. (2006) 393(Pt 1):7–20. 10.1042/BJ20051578 16336196 PMC1383660

[49] Faulkner JL Bruder-Nascimento T Belin de Chantemele EJ. The regulation of aldosterone secretion by leptin: implications in obesity-related cardiovascular disease. Curr Opin Nephrol Hypertens. (2018) 27:63–9. 10.1097/MNH.0000000000000384 29135585 PMC6053669

[50] Xie D Bollag WB. Obesity, hypertension and aldosterone: is leptin the link? J Endocrinol. (2016) 230:F7-F11. 10.1530/JOE-16-0160 27252389 PMC8350967

[51] Vatutin NT Shevelok AN. Relationship between blood aldosterone and somatometric parameters in patients with chronic heart failure and preserved ejection fraction of left ventricle. Klin Med. (2016) 94:265–9. 28957604

[52] Olivier A Pitt B Girerd N Lamiral Z Machu JL McMurray JJV. Effect of eplerenone in patients with heart failure and reduced ejection fraction: potential effect modification by abdominal obesity. Insight from the EMPHASIS-HF trial. Eur J Heart Fail. (2017) 19:1186–97. 10.1002/ejhf.792 28303624

[53] Kotlyar E Vita JA Winter MR Awtry EH Siwik DA Keaney JF Jr. The relationship between aldosterone, oxidative stress, and inflammation in chronic, stable human heart failure. J Card Fail. (2006) 12:122–7. 10.1016/j.cardfail.2005.08.005 16520260

[54] Shieh FK Kotlyar E Sam F. Aldosterone and cardiovascular remodelling: focus on myocardial failure. J Renin Angiotensin Aldosterone Syst. (2004) 5:3–13. 10.3317/jraas.2004.005 15136967

[55] Na T Dai DZ Tang XY Dai Y. Upregulation of leptin pathway correlates with abnormal expression of SERCA2a, phospholamban and the endothelin pathway in heart failure and reversal by CPU86017. Naunyn Schmiedebergs Arch Pharmacol. (2007) 375:39–49. 10.1007/s00210-007-0134-1 17287947

[56] Van den Bergh A Vanderper A Vangheluwe P Desjardins F Nevelsteen I Verreth W Dyslipidaemia in type II diabetic mice does not aggravate contractile impairment but increases ventricular stiffness. Cardiovasc Res. (2008) 77:371–9. 10.1093/cvr/cvm001 18006491

[57] Sam F Walsh K. What can adiponectin say about left ventricular function? Heart. (2010) 96:331–2. 10.1136/hrt.2009.178590 19934101 PMC3673540

[58] Engeli S Feldpausch M Gorzelniak K Hartwig F Heintze U Janke J. Association between adiponectin and mediators of inflammation in obese women. Diabetes. (2003) 52:942–7. 10.2337/diabetes.52.4.942 12663465

[59] Hong SJ Park CG Seo HS Oh DJ Ro YM. Associations among plasma adiponectin, hypertension, left ventricular diastolic function and left ventricular mass index. Blood Press. (2004) 13:236–42. 10.1080/08037050410021397 15581338

[60] Francisco C Neves JS Falcao-Pires I Leite-Moreira A. Can Adiponectin Help us to Target Diastolic Dysfunction? Cardiovasc Drugs Ther. (2016) 30:635–44. 10.1007/s10557-016-6694-x 27757724

[61] Ouchi N Kihara S Arita Y Maeda K Kuriyama H Okamoto Y. Novel modulator for endothelial adhesion molecules: adipocyte-derived plasma protein adiponectin. Circulation. (1999) 100:2473–6. 10.1161/01.CIR.100.25.2473 10604883

[62] Essick EE Wilson RM Pimentel DR Shimano M Baid S Ouchi N. Adiponectin modulates oxidative stress-induced autophagy in cardiomyocytes. PLoS ONE. (2013) 8:e68697. 10.1371/journal.pone.0068697 23894332 PMC3716763

[63] Essick EE Ouchi N Wilson RM Ohashi K Ghobrial J Shibata R. Adiponectin mediates cardioprotection in oxidative stress-induced cardiac myocyte remodeling. Am J Physiol Heart Circ Physiol. (2011) 301:H984–93. 10.1152/ajpheart.00428.2011 21666115 PMC3191107

[64] Shibata R Ouchi N Ito M Kihara S Shiojima I Pimentel DR. Adiponectin-mediated modulation of hypertrophic signals in the heart. Nat Med. (2004) 10:1384–9. 10.1038/nm1137 15558058 PMC2828675

[65] Ouchi N Kobayashi H Kihara S Kumada M Sato K Inoue T. Adiponectin stimulates angiogenesis by promoting cross-talk between AMP-activated protein kinase and Akt signaling in endothelial cells. J Biol Chem. (2004) 279:1304–9. 10.1074/jbc.M310389200 14557259 PMC4374490

[66] Frankel DS Vasan RS D'Agostino RB Sr. Benjamin EJ Levy D Wang TJ. Resistin, adiponectin, and risk of heart failure the Framingham offspring study. J Am Coll Cardiol. (2009) 53:754–62. 10.1016/j.jacc.2008.07.073 19245965 PMC2676793

[67] Kistorp C Faber J Galatius S Gustafsson F Frystyk J Flyvbjerg A. Plasma adiponectin, body mass index, and mortality in patients with chronic heart failure. Circulation. (2005) 112:1756–62. 10.1161/CIRCULATIONAHA.104.530972 16157772

[68] George J Patal S Wexler D Sharabi Y Peleg E Kamari Y. Circulating adiponectin concentrations in patients with congestive heart failure. Heart. (2006) 92:1420–4. 10.1136/hrt.2005.083345 16621874 PMC1861042

[69] Tamura T Furukawa Y Taniguchi R Sato Y Ono K Horiuchi H. Serum adiponectin level as an independent predictor of mortality in patients with congestive heart failure. Circ J. (2007) 71:623–30. 10.1253/circj.71.623 17456982

[70] Antoniades C Antonopoulos AS Tousoulis D Stefanadis C. Adiponectin: from obesity to cardiovascular disease. Obes Rev. (2009) 10:269–79. 10.1111/j.1467-789X.2009.00571.x 19389061

[71] Behre CJ. Adiponectin: saving the starved and the overfed. Med Hypotheses. (2007) 69:1290–2. 10.1016/j.mehy.2007.02.044 17509773

[72] Biolo A Shibata R Ouchi N Kihara S Sonoda M Walsh K. Determinants of adiponectin levels in patients with chronic systolic heart failure. Am J Cardiol. (2010) 105:1147–52. 10.1016/j.amjcard.2009.12.015 20381668 PMC2854672

[73] Norvik JV Schirmer H Ytrehus K Jenssen TG Zykova SN Eggen AE. Low adiponectin is associated with diastolic dysfunction in women: a cross-sectional study from the Tromso Study. BMC Cardiovasc Disord. (2017) 17:79. 10.1186/s12872-017-0509-2 28292262 PMC5351172

[74] Sam F Duhaney TA Sato K Wilson RM Ohashi K Sono-Romanelli S. Adiponectin deficiency, diastolic dysfunction, and diastolic heart failure. Endocrinology. (2010) 151:322–31. 10.1210/en.2009-0806 19850745 PMC2803144

[75] Tanaka K Wilson RM Essick EE Duffen JL Scherer PE Ouchi N. Effects of adiponectin on calcium-handling proteins in heart failure with preserved ejection fraction. Circ Heart Fail. (2014) 7:976–85. 10.1161/CIRCHEARTFAILURE.114.001279 25149095 PMC4241144

[76] Fuster JJ Ouchi N Gokce N Walsh K. Obesity-induced changes in adipose tissue microenvironment and their impact on cardiovascular disease. Circ Res. (2016) 118:1786–807. 10.1161/CIRCRESAHA.115.306885 27230642 PMC4887147

[77] Schiattarella GG Altamirano F Tong D French KM Villalobos E Kim SY. Nitrosative stress drives heart failure with preserved ejection fraction. Nature. (2019) 568:351–6. 10.1038/s41586-019-1100-z 30971818 PMC6635957

[78] Chirinos JA Zamani P. The Nitrate-Nitrite-NO Pathway and its implications for heart failure and preserved ejection fraction. Curr Heart Fail Rep. (2016) 13:47–59. 10.1007/s11897-016-0277-9 26792295 PMC4755323

[79] Alvarez P Briasoulis A. Immune modulation in heart failure: the promise of novel biologics. Curr Treat Options Cardiovasc Med. (2018) 20:26. 10.1007/s11936-018-0617-z 29541873

[80] Hulsmans M Sager HB Roh JD Valero-Munoz M Houstis NE Iwamoto Y. Cardiac macrophages promote diastolic dysfunction. J Exp Med. (2018) 215:423–40. 10.1084/jem.20171274 29339450 PMC5789416

[81] Van Tassell BW Trankle CR Canada JM Carbone S Buckley L Kadariya D. IL-1 blockade in patients with heart failure with preserved ejection fraction. Circ Heart Fail. (2018) 11:e005036. 10.1161/CIRCHEARTFAILURE.118.005036 30354558 PMC6545106

[82] Van Tassell BW Buckley LF Carbone S Trankle CR Canada JM Dixon DL. Interleukin-1 blockade in heart failure with preserved ejection fraction: rationale and design of the Diastolic Heart Failure Anakinra Response Trial 2 (D-HART2). Clin Cardiol. (2017) 40:626–32. 10.1002/clc.22719 28475816 PMC5744484

[83] Upadhya B Haykowsky MJ Eggebeen J Kitzman DW. Exercise intolerance in heart failure with preserved ejection fraction: more than a heart problem. J Geriatr Cardiol. (2015) 12:294–304. 10.1007/s11897-015-0257-5 26089855 PMC4460174

[84] Houstis NE Eisman AS Pappagianopoulos PP Wooster L Bailey CS Wagner PD. Exercise intolerance in heart failure with preserved ejection fraction: diagnosing and ranking its causes using personalized O2 pathway analysis. Circulation. (2018) 137:148–61. 10.1161/CIRCULATIONAHA.117.029058 28993402 PMC5760316

[85] Wolfel EE. Exploring the mechanisms of exercise intolerance in patients with HFpEF: are we too “Cardiocentric?”. JACC Heart Fail. (2016) 4:646–8. 10.1016/j.jchf.2016.06.002 27469629

[86] Carbone S Canada JM Buckley LF Trankle CR Dixon DL Buzzetti R. Obesity contributes to exercise intolerance in heart failure with preserved ejection fraction. J Am Coll Cardiol. (2016) 68:2487–8. 10.1016/j.jacc.2016.08.072 27908355 PMC5748881

[87] Haykowsky MJ Nicklas BJ Brubaker PH Hundley WG Brinkley TE Upadhya B. Regional adipose distribution and its relationship to exercise intolerance in older obese patients who have heart failure with preserved ejection fraction. JACC Heart Fail. (2018) 6:640–9. 10.1016/j.jchf.2018.06.002 30007558 PMC6086374

[88] Kokkinos P Faselis C Franklin B Lavie CJ Sidossis L Moore H. Cardiorespiratory fitness, body mass index and heart failure incidence. Eur J Heart Fail. (2019) 21:436–44. 10.1002/ejhf.1433 30779281

[89] Kitzman DW Brubaker PH Morgan TM Stewart KP Little WC. Exercise training in older patients with heart failure and preserved ejection fraction: a randomized, controlled, single-blind trial. Circ Heart Fail. (2010) 3:659–67. 10.1161/CIRCHEARTFAILURE.110.958785 20852060 PMC3065299

[90] Kitzman DW Brubaker PH Herrington DM Morgan TM Stewart KP Hundley WG. Effect of endurance exercise training on endothelial function and arterial stiffness in older patients with heart failure and preserved ejection fraction: a randomized, controlled, single-blind trial. J Am Coll Cardiol. (2013) 62:584–92. 10.1016/j.jacc.2013.04.033 23665370 PMC3740089

[91] Edelmann F Gelbrich G Dungen HD Frohling S Wachter R Stahrenberg R. Exercise training improves exercise capacity and diastolic function in patients with heart failure with preserved ejection fraction: results of the Ex-DHF (Exercise training in Diastolic Heart Failure) pilot study. J Am Coll Cardiol. (2011) 58:1780–91. 10.1016/j.jacc.2011.06.054 21996391

[92] Kitzman DW Brubaker P Morgan T Haykowsky M Hundley G Kraus WE. Effect of Caloric restriction or aerobic exercise training on peak oxygen consumption and quality of life in obese older patients with heart failure with preserved ejection fraction: a randomized clinical trial. JAMA. (2016) 315:36–46. 10.1001/jama.2015.17346 26746456 PMC4787295

[93] Prenner SB Mather PJ. Obesity and heart failure with preserved ejection fraction: a growing problem. Trends Cardiovasc Med. (2018) 28:322–7. 10.1016/j.tcm.2017.12.003 29305040

[94] Lavie CJ Alpert MA Arena R Mehra MR Milani RV Ventura HO. Impact of obesity and the obesity paradox on prevalence and prognosis in heart failure. JACC Heart Fail. (2013) 1:93–102. 10.1016/j.jchf.2013.01.006 24621833

[95] Oreopoulos A Padwal R Kalantar-Zadeh K Fonarow GC Norris CM McAlister FA. Body mass index and mortality in heart failure: a meta-analysis. Am Heart J. (2008) 156:13–22. 10.1016/j.ahj.2008.02.014 18585492

[96] Horwich TB Fonarow GC Hamilton MA MacLellan WR Woo MA Tillisch JH. The relationship between obesity and mortality in patients with heart failure. J Am Coll Cardiol. (2001) 38:789–95. 10.1016/S0735-1097(01)01448-6 11527635

[97] Lissin LW Gauri AJ Froelicher VF Ghayoumi A Myers J Giacommini J. The prognostic value of body mass index and standard exercise testing in male veterans with congestive heart failure. J Card Fail. (2002) 8:206–15. 10.1054/jcaf.2002.126812 12397568

[98] Powell-Wiley TM Ngwa J Kebede S Lu D Schulte PJ Bhatt DL. Impact of body mass index on heart failure by race/ethnicity from the get with The Guidelines-Heart Failure (GWTG-HF) Registry. JACC Heart Fail. (2018) 6:233–42. 10.1016/j.jchf.2017.11.011 29428434 PMC5834392

[99] Padwal R McAlister FA McMurray JJ Cowie MR Rich M Pocock S. The obesity paradox in heart failure patients with preserved versus reduced ejection fraction: a meta-analysis of individual patient data. Int J Obes. (2014) 38:1110–4. 10.1038/ijo.2013.203 24173404

[100] Kenchaiah S Pocock SJ Wang D Finn PV Zornoff LA Skali H. Body mass index and prognosis in patients with chronic heart failure: insights from the Candesartan in Heart failure: Assessment of Reduction in Mortality and morbidity (CHARM) program. Circulation. (2007) 116:627–36. 10.1161/CIRCULATIONAHA.106.679779 17638930

[101] Kapoor JR Heidenreich PA. Obesity and survival in patients with heart failure and preserved systolic function: a U-shaped relationship. Am Heart J. (2010) 159:75–80. 10.1016/j.ahj.2009.10.026 20102870

[102] Adamopoulos C Meyer P Desai RV Karatzidou K Ovalle F White M. Absence of obesity paradox in patients with chronic heart failure and diabetes mellitus: a propensity-matched study. Eur J Heart Fail. (2011) 13:200–6. 10.1093/eurjhf/hfq159 20930001 PMC3025667

[103] Pozzo J Fournier P Lairez O Vervueren PL Delmas C Elbaz M. Obesity paradox: origin and best way to assess severity in patients with systolic HF. Obesity. (2015) 23:2002–8. 10.1002/oby.21216 26337500

[104] Vest AR Wu Y Hachamovitch R Young JB Cho L. The heart failure overweight/obesity survival paradox: the missing sex link. JACC Heart Fail. (2015) 3:917–26. 10.1016/j.jchf.2015.06.009 26454846

[105] Horwich TB Fonarow GC Clark AL. Obesity and the obesity paradox in heart failure. Prog Cardiovasc Dis. (2018) 61:151–6. 10.1016/j.pcad.2018.05.005 29852198

[106] Nagarajan V Kohan L Holland E Keeley EC Mazimba S. Obesity paradox in heart failure: a heavy matter. ESC Heart Fail. (2016) 3:227–34. 10.1002/ehf2.12120 27867523 PMC5107969

[107] Carbone S Lavie CJ Arena R. Obesity and heart failure: focus on the obesity paradox. Mayo Clin Proc. (2017) 92:266–79. 10.1016/j.mayocp.2016.11.001 28109619

[108] Tadic M Cuspidi C Obesity and heart failure with preserved ejection fraction: a paradox or something else? Heart Fail Rev. (2019) 24:379–85. 10.1007/s10741-018-09766-x 30610456

[109] Wang TJ. The obesity paradox in heart failure: weighing the evidence. J Am Coll Cardiol. (2014) 64:2750–2. 10.1016/j.jacc.2014.09.068 25541127

[110] Gruden G Landi A Bruno G. Natriuretic peptides, heart, and adipose tissue: new findings and future developments for diabetes research. Diabetes Care. (2014) 37:2899–908. 10.2337/dc14-0669 25342830

[111] Stingo AJ Clavell AL Heublein DM Wei CM Pittelkow MR Burnett JC Jr. Presence of C-type natriuretic peptide in cultured human endothelial cells and plasma. Am J Physiol. (1992) 263(4 Pt 2):H1318–21. 10.1152/ajpheart.1992.263.4.H1318 1384363

[112] Potter LR Abbey-Hosch S Dickey DM. Natriuretic peptides, their receptors, and cyclic guanosine monophosphate-dependent signaling functions. Endocr Rev. (2006) 27:47–72. 10.1210/er.2005-0014 16291870

[113] Collins S. A heart-adipose tissue connection in the regulation of energy metabolism. Nat Rev Endocrinol. (2014) 10:157–63. 10.1038/nrendo.2013.234 24296515

[114] Palmer BF Clegg DJ. An emerging role of natriuretic peptides: igniting the fat furnace to fuel and warm the heart. Mayo Clin Proc. (2015) 90:1666–78. 10.1016/j.mayocp.2015.08.006 26518101

[115] Zois NE Bartels ED Hunter I Kousholt BS Olsen LH Goetze JP. Natriuretic peptides in cardiometabolic regulation and disease. Nat Rev Cardiol. (2014) 11:403–12. 10.1038/nrcardio.2014.64 24820868

[116] McKie PM Burnett JC Jr. NT-proBNP: The gold standard biomarker in heart failure. J Am Coll Cardiol. (2016) 68:2437–9. 10.1016/j.jacc.2016.10.001 27908348

[117] Zile MR Claggett BL Prescott MF McMurray JJ Packer M Rouleau JL. Prognostic implications of changes in N-terminal pro-B-Type natriuretic peptide in patients with heart failure. J Am Coll Cardiol. (2016) 68:2425–36. 10.1016/j.jacc.2016.09.931 27908347

[118] Wang TJ Larson MG Levy D Benjamin EJ Leip EP Omland T. Plasma natriuretic peptide levels and the risk of cardiovascular events and death. N Engl J Med. (2004) 350:655–63. 10.1056/NEJMoa031994 14960742

[119] Fonarow GC Peacock WF Phillips CO Givertz MM Lopatin M. Admission B-type natriuretic peptide levels and in-hospital mortality in acute decompensated heart failure. J Am Coll Cardiol. (2007) 49:1943–50. 10.1016/j.jacc.2007.02.037 17498579

[120] O'Donoghue M Chen A Baggish AL Anwaruddin S Krauser DG Tung R. The effects of ejection fraction on N-terminal ProBNP and BNP levels in patients with acute CHF: analysis from the ProBNP Investigation of Dyspnea in the Emergency Department (PRIDE) study. J Card Fail. (2005) 11(5 Suppl.):S9–14. 10.1016/j.cardfail.2005.04.011 15948094

[121] Januzzi JL Jr. Natriuretic peptides, ejection fraction, and prognosis: parsing the phenotypes of heart failure. J Am Coll Cardiol. (2013) 61:1507–9. 10.1016/j.jacc.2013.01.039 23500284

[122] Stavrakis S Pakala A Thomas J Chaudhry MA Thadani U. Obesity, brain natriuretic peptide levels and mortality in patients hospitalized with heart failure and preserved left ventricular systolic function. Am J Med Sci. (2013) 345:211–7. 10.1097/MAJ.0b013e318271c012 23422653

[123] Buckley LF Canada JM Del Buono MG Carbone S Trankle CR Billingsley H Low NT-proBNP levels in overweight and obese patients do not rule out a diagnosis of heart failure with preserved ejection fraction. ESC Heart Fail. (2018) 5:372–8. 10.1002/ehf2.12235 PMC5880665 29345112

[124] Daniels LB Clopton P Bhalla V Krishnaswamy P Nowak RM McCord J How obesity affects the cut-points for B-type natriuretic peptide in the diagnosis of acute heart failure. Results from the Breathing Not Properly Multinational Study. Am Heart J. (2006) 151:999–1005. 10.1016/j.ahj.2005.10.011 16644321

[125] Zhang H Thoonen R Yao V Buys ES Popovich J Su YR. Regulation of B-type natriuretic peptide synthesis by insulin in obesity in male mice. Exp Physiol. (2016) 101:113–23. 10.1113/EP085091 26446173

[126] Cabiati M Raucci S Liistro T Belcastro E Prescimone T Caselli C. Impact of obesity on the expression profile of natriuretic peptide system in a rat experimental model. PLoS ONE. (2013) 8:e72959. 10.1371/journal.pone.0072959 24009719 PMC3756951

[127] Bartels ED Nielsen JM Bisgaard LS Goetze JP Nielsen LB. Decreased expression of natriuretic peptides associated with lipid accumulation in cardiac ventricle of obese mice. Endocrinology. (2010) 151:5218–25. 10.1210/en.2010-0355 20844006

[128] Nishimura M Brann A Chang KW Maisel AS. The confounding effects of non-cardiac pathologies on the interpretation of cardiac biomarkers. Curr Heart Fail Rep. (2018) 15:239–49. 10.1007/s11897-018-0398-4 29987498

[129] Gentili A Frangione MR Albini E Vacca C Ricci MA De VS. Modulation of natriuretic peptide receptors in human adipose tissue: molecular mechanisms behind the “natriuretic handicap” in morbidly obese patients. Transl Res. (2017) 186:52–61. 10.1016/j.trsl.2017.06.001 28651075

[130] Shah Z Wiley M Sridhar AM Masoomi R Biria M Lakkireddy D. Inverse correlation of venous brain natriuretic peptide levels with body mass index is due to decreased production. Cardiology. (2017) 137:159–66. 10.1159/000464111 28391273

[131] Mizuno Y Harada E Katoh D Kashiwagi Y Morikawa Y Nakagawa H. Cardiac production of B-type natriuretic peptide is inversely related to the plasma level of free fatty acids in obese individuals - possible involvement of the insulin resistance -. Endocr J. (2013) 60:87–95. 10.1507/endocrj.EJ12-0239 23006812

[132] Plante E Menaouar A Danalache BA Broderick TL Jankowski M Gutkowska J. Treatment with brain natriuretic peptide prevents the development of cardiac dysfunction in obese diabetic db/db mice. Diabetologia. (2014) 57:1257–67. 10.1007/s00125-014-3201-4 24595856

[133] Khan AM Cheng S Magnusson M Larson MG Newton-Cheh C McCabe EL. Cardiac natriuretic peptides, obesity, and insulin resistance: evidence from two community-based studies. J Clin Endocrinol Metab. (2011) 96:3242–9. 10.1210/jc.2011-1182 21849523 PMC3200240

[134] Moro C. Targeting cardiac natriuretic peptides in the therapy of diabetes and obesity. Expert Opin Ther Targets. (2016) 20:1445–52. 10.1080/14728222.2016.1254198 27786597

[135] Volpe M Carnovali M Mastromarino V. The natriuretic peptides system in the pathophysiology of heart failure: from molecular basis to treatment. Clin Sci. (2016) 130:57–77. 10.1042/CS20150469 26637405 PMC5233571

[136] Standeven KF Hess K Carter AM Rice GI Cordell PA Balmforth AJ. Neprilysin, obesity and the metabolic syndrome. Int J Obes. (2011) 35:1031–40. 10.1038/ijo.2010.227 21042321 PMC3040694

[137] Goliasch G Pavo N Zotter-Tufaro C Kammerlander A Duca F Mascherbauer J Soluble neprilysin does not correlate with outcome in heart failure with preserved ejection fraction. Eur J Heart Fail. (2016) 18:89–93. 10.1002/ejhf.435 26725876

[138] Weber M Hamm C. Role of B-type natriuretic peptide (BNP) and NT-proBNP in clinical routine. Heart. (2006) 92:843–9. 10.1136/hrt.2005.071233 16698841 PMC1860679

[139] Solomon SD Zile M Pieske B Voors A Shah A Kraigher-Krainer E. The angiotensin receptor neprilysin inhibitor LCZ696 in heart failure with preserved ejection fraction: a phase 2 double-blind randomised controlled trial. Lancet. (2012) 380:1387–95. 10.1016/S0140-6736(12)61227-6 22932717

[140] Solomon SD Rizkala AR Gong J Wang W Anand IS Ge J. Angiotensin receptor neprilysin inhibition in heart failure with preserved ejection fraction: rationale and design of the PARAGON-HF Trial. JACC Heart Fail. (2017) 5:471–82. 10.1016/j.jchf.2017.04.013 28662936

[141] Thoonen R Hindle AG Scherrer-Crosbie M. Brown adipose tissue: The heat is on the heart. Am J Physiol Heart Circ Physiol. (2016) 310:H1592–605. 10.1152/ajpheart.00698.2015 27084389 PMC6345214

[142] Lee P Swarbrick MM Ho KK. Brown adipose tissue in adult humans: a metabolic renaissance. Endocr Rev. (2013) 34:413–38. 10.1210/er.2012-1081 23550082

[143] Bartelt A Heeren J. Adipose tissue browning and metabolic health. Nat Rev Endocrinol. (2014) 10:24–36. 10.1038/nrendo.2013.204 24146030

[144] Bordicchia M Liu D Amri EZ Ailhaud G Dessi-Fulgheri P Zhang C. Cardiac natriuretic peptides act via p38 MAPK to induce the brown fat thermogenic program in mouse and human adipocytes. J Clin Invest. (2012) 122:1022–36. 10.1172/JCI59701 22307324 PMC3287224

[145] Sengenes C Berlan M De Glisezinski I Lafontan M Galitzky J. Natriuretic peptides: a new lipolytic pathway in human adipocytes. FASEB J. (2000) 14:1345–51. 10.1096/fasebj.14.10.1345 10877827

[146] Valero-Munoz M Li S Wilson RM Hulsmans M Aprahamian T Fuster JJ. Heart failure with preserved ejection fraction induces beiging in adipose tissue. Circ Heart Fail. (2016) 9:e002724. 10.1161/CIRCHEARTFAILURE.115.002724 26721917 PMC4698901

[147] Whittle AJ Vidal-Puig A. NPs – heart hormones that regulate brown fat? J Clin Invest. (2012) 122:804–7. 10.1172/JCI62595 22307322 PMC3287241

[148] Matsukawa N Grzesik WJ Takahashi N Pandey KN Pang S Yamauchi M. The natriuretic peptide clearance receptor locally modulates the physiological effects of the natriuretic peptide system. Proc Natl Acad Sci USA. (1999) 96:7403–8. 10.1073/pnas.96.13.7403 10377427 PMC22098

[149] Lafontan M Moro C Berlan M Crampes F Sengenes C Galitzky J. Control of lipolysis by natriuretic peptides and cyclic GMP. Trends Endocrinol Metab. (2008) 19:130–7. 10.1016/j.tem.2007.11.006 18337116

[150] Austin S St-Pierre J. PGC1alpha and mitochondrial metabolism–emerging concepts and relevance in ageing and neurodegenerative disorders. J Cell Sci. (2012) 125(Pt 21):4963–71. 10.1242/jcs.113662 23277535

[151] Kooijman S van den Heuvel JK Rensen PCN. Neuronal control of brown fat activity. Trends Endocrinol Metab. (2015) 26:657–68. 10.1016/j.tem.2015.09.008 26482876

[152] Cannon B Nedergaard J. Brown adipose tissue: function and physiological significance. Physiol Rev. (2004) 84:277–359. 10.1152/physrev.00015.2003 14715917

[153] Collins S Sarzani R Bordicchia M. Coordinate control of adipose 'browning' and energy expenditure by beta-adrenergic and natriuretic peptide signalling. Int J Obes Suppl. (2014) 4(Suppl. 1):S17–20. 10.1038/ijosup.2014.6 27152160 PMC4850585

[154] Wang TJ Larson MG Levy D Benjamin EJ Leip EP Wilson PW. Impact of obesity on plasma natriuretic peptide levels. Circulation. (2004) 109:594–600. 10.1161/01.CIR.0000112582.16683.EA 14769680

[155] Wang TJ Larson MG Keyes MJ Levy D Benjamin EJ Vasan RS. Association of plasma natriuretic peptide levels with metabolic risk factors in ambulatory individuals. Circulation. (2007) 115:1345–53. 10.1161/CIRCULATIONAHA.106.655142 17339551

[156] Kovacova Z Tharp WG Liu D Wei W Xie H Collins S. Adipose tissue natriuretic peptide receptor expression is related to insulin sensitivity in obesity and diabetes. Obesity. (2016) 24:820–8. 10.1002/oby.21418 26887289 PMC5067565

[157] Nakatsuji H Maeda N Hibuse T Hiuge A Hirata A Kuroda Y. Reciprocal regulation of natriuretic peptide receptors by insulin in adipose cells. Biochem Biophys Res Commun. (2010) 392:100–5. 10.1016/j.bbrc.2010.01.008 20059960

[158] Bordicchia M Ceresiani M Pavani M Minardi D Polito M Wabitsch M. Insulin/glucose induces natriuretic peptide clearance receptor in human adipocytes: a metabolic link with the cardiac natriuretic pathway. Am J Physiol Regul Integr Comp Physiol. (2016) 311:R104–14. 10.1152/ajpregu.00499.2015 27101299

[159] Coue M Barquissau V Morigny P Louche K Lefort C Mairal A. Natriuretic peptides promote glucose uptake in a cGMP-dependent manner in human adipocytes. Sci Rep. (2018) 8:1097. 10.1038/s41598-018-19619-0 29348496 PMC5773662

[160] Moro C Klimcakova E Lolmede K Berlan M Lafontan M Stich V. Atrial natriuretic peptide inhibits the production of adipokines and cytokines linked to inflammation and insulin resistance in human subcutaneous adipose tissue. Diabetologia. (2007) 50:1038–47. 10.1007/s00125-007-0614-3 17318625

[161] Fain JN Kanu A Bahouth SW Cowan GS Lloyd HM. Inhibition of leptin release by atrial natriuretic peptide (ANP) in human adipocytes. Biochem Pharmacol. (2003) 65:1883–8. 10.1016/S0006-2952(03)00154-0 12781340

[162] Melenovsky V Kotrc M Borlaug BA Marek T Kovar J Malek I. Relationships between right ventricular function, body composition, and prognosis in advanced heart failure. J Am Coll Cardiol. (2013) 62:1660–70. 10.1016/j.jacc.2013.06.046 23916933

[163] Birkenfeld AL Boschmann M Engeli S Moro C Arafat AM Luft FC. Atrial natriuretic peptide and adiponectin interactions in man. PLoS ONE. (2012) 7:e43238. 10.1371/journal.pone.0043238 22916229 PMC3420865

[164] Tsukamoto O Fujita M Kato M Yamazaki S Asano Y Ogai A. Natriuretic peptides enhance the production of adiponectin in human adipocytes and in patients with chronic heart failure. J Am Coll Cardiol. (2009) 53:2070–7. 10.1016/j.jacc.2009.02.038 19477358

[165] Neeland IJ Winders BR Ayers CR Das SR Chang AY Berry JD. Higher natriuretic peptide levels associate with a favorable adipose tissue distribution profile. J Am Coll Cardiol. (2013) 62:752–60. 10.1016/j.jacc.2013.03.038 23602771 PMC3857334

[166] Karas MG Benkeser D Arnold AM Bartz TM Djousse L Mukamal KJ. Relations of plasma total and high-molecular-weight adiponectin to new-onset heart failure in adults >/=65 years of age (from the Cardiovascular Health study). Am J Cardiol. (2014) 113:328–34. 10.1016/j.amjcard.2013.09.027 24169012 PMC3968249

